# The clinical outcome and microbiological profile of bone-anchored hearing systems (BAHS) with different abutment topographies: a prospective pilot study

**DOI:** 10.1007/s00405-018-4946-z

**Published:** 2018-04-05

**Authors:** Margarita Trobos, Martin Lars Johansson, Sofia Jonhede, Hanna Peters, Maria Hoffman, Omar Omar, Peter Thomsen, Malou Hultcrantz

**Affiliations:** 1 Department of Biomaterials, Institute of Clinical Sciences, Sahlgrenska Academy, University of Gothenburg, P.O. Box 412, 405 30 Gothenburg, Sweden; 2 Oticon Medical AB, Askim, Sweden; 3 Department of Otorhinolaryngology, Karolinska University Hospital, Stockholm, Sweden

**Keywords:** Abutment, Bacteria, BAHS, Bone-anchored hearing system, Skin, *Staphylococcus*

## Abstract

**Purpose:**

In this prospective clinical pilot study, abutments with different topologies (machined versus polished) were compared with respect to the clinical outcome and the microbiological profile. Furthermore, three different sampling methods (retrieval of abutment, collection of peri-abutment exudate using paper-points, and a small peri-abutment soft-tissue biopsy) were evaluated for the identification and quantification of colonising bacteria.

**Methods:**

Twelve patients, seven with machined abutment and five with polished abutment, were included in the analysis. Three different sampling procedures were employed for the identification and quantification of colonising bacteria from baseline up to 12 months, using quantitative culturing. Clinical outcome measures (Holgers score, hygiene, pain, numbness and implant stability) were investigated.

**Results:**

The clinical parameters, and total viable bacteria per abutment or in tissue biopsies did not differ significantly between the polished and machined abutments. The total CFU/mm^2^ abutment and CFU/peri-abutment fluid space of anaerobes, aerobes and staphylococci were significantly higher for the polished abutment. Anaerobic bacteria were detected in the tissue biopsies before BAHS implantation. Anaerobes and *Staphylococcus* spp. were detected in all three compartments after BAHS installation. For most patients (10/12), the same staphylococcal species were found in at least two of the three compartments at the same time-point. The common skin coloniser *Staphylococcus epidermidis* was identified in all patients but one (11/12), whereas the pathogen *Staphylococcus aureus* was isolated in five of the patients. Several associations between clinical and microbiological parameters were found.

**Conclusions:**

There was no difference in the clinical outcome with the use of polished versus machined abutment at 3 and 12 months after implantation. The present pilot trial largely confirmed a suitable study design, sampling and analytical methodology to determine the effects of modified BAHS abutment properties.

**Level of evidence:**

2. Controlled prospective comparative study.

**Electronic supplementary material:**

The online version of this article (10.1007/s00405-018-4946-z) contains supplementary material, which is available to authorized users.

## Introduction

The percutaneous bone-anchored hearing system (BAHS) has been used clinically for over 30 years and is the most commonly used system for patients with conductive or mixed hearing losses. The long-term success rate is high, with a low rate of major complications. Nevertheless, adverse skin reactions continue to be common complications [1, 2]. Most reactions are mild to moderate, but still cause inconvenience for patients and additional costs for the health care system, supporting the strong need to optimise the conditions of the abutment–skin interface.

After surgical installation of implants, inflammation followed by tissue repair are the two major biological events elicited in the bone and soft tissue in immediate contact with the components of the BAHS. The detailed mechanisms of these responses such as how they are affected by the host, local tissue conditions, surgical protocols and material properties, have largely been derived from experimental studies and other applications (e.g. oral implants) [3–5]. A key process, ultimately resulting in a stable fixation of the BAHS, is the osseointegration of the fixture in the temporal bone. In contrast, the nature of a long-term and optimal soft tissue adaptation to the abutment material, penetrating the skin, is incompletely understood. During adverse tissue responses to BAHS, the surrounding immune cells and microorganisms may be localised in several different compartments: on the surface of the abutment, in the exudate around the abutment, and in the surrounding connective tissue [6–9]. A major reason why the structural and functional adaptation of the implanted material to the bone and soft tissue is not trivial, is the concept “race for the surface” between host cells and microorganisms [10]. Furthermore, the presence of a foreign material reduces the minimum dose needed to cause infection by 10,000-fold [11]. An aggravating factor for percutaneous implants is the breach of the skin barrier, making the entire system vulnerable for colonisation of microorganisms.

The physicochemical and topographical properties of biomaterials are key variables for achieving a desired host tissue response [12]. Recently, hydroxyapatite (HA)-coated titanium abutments were introduced clinically for BAHS [13]. The intention was to achieve integration between the abutment material and the dermal tissue, thereby reducing the pocket formation and bacterial colonisation on the abutment surface. Although dermal adherence has been demonstrated experimentally, the short- and long-term clinical benefits have varied [4, 13, 14]. Moreover, shear and strain forces in the soft tissue may induce adverse inflammatory responses [15]. Here, we hypothesise that an ultra-smooth abutment, achieved by electropolishing, will prevent adhesion and integration allowing the soft tissue to move freely around the abutment, counteracting the mechanical discrepancy. Electropolishing is a process for material removal acting selectively on micro-rough areas resulting in a microscopically smooth and shiny surface while maintaining the macroscopic structure intact [16].

Studies evaluating the role of the microbial component for the tissue response and outcome for BAHS are limited. Using electron microscopy, fungi and bacteria have been observed on abutments obtained from humans, both free on the surface, in the form of biofilm and internalised in macrophages and granulocytes [6–8]. In a clinical study, the presence of *Staphylococcus aureus* in the soft tissue adjacent to the abutment was associated with a clinical manifestation of skin irritation [17]. Sebaceous glands connected to hair follicles secrete sebum, acting as an antibacterial shield that protects and lubricates the skin and hair. The relatively anoxic conditions of sebaceous glands can however promote the growth of facultative anaerobes like *Propionibacterium acnes* [18]. Furthermore, the skin microbiota is dependent on the body site, with the lowest bacterial diversity found in sebaceous sites like the retro-auricular crease (behind the ear) with 15 phylotypes [19, 20]. *Propionibacterium* spp., *Staphylococcus* spp. and *Corynebacterium* spp. predominate in sebaceous sites of the skin in culture-based [21] and genomic [20] studies. However, details on how the microflora of the retro-auricular crease/scalp changes in response to percutaneous abutments connected to osseointegrated implants are unknown.

The aims of this prospective clinical pilot study were to: (1) evaluate and compare abutments with different topologies (machined versus polished) with respect to the clinical outcome and the microbiological profile up to 1 year, and (2) evaluate three different sampling methods (retrieval of abutment, collection of peri-abutment exudate using paper-points, and small peri-abutment soft-tissue biopsy) for the identification and quantification of colonising bacteria.

## Materials and methods

### Ethics

The study was performed in consistency with the Declaration of Helsinki (Washington 2002), ISO 14155:2011, approved by the Regional Ethical Review Board of Stockholm, Sweden (2014/1566-31/2) and registered at clinicaltrials.gov (NCT02304692).

### Patients and implants

**Fig. 1:**
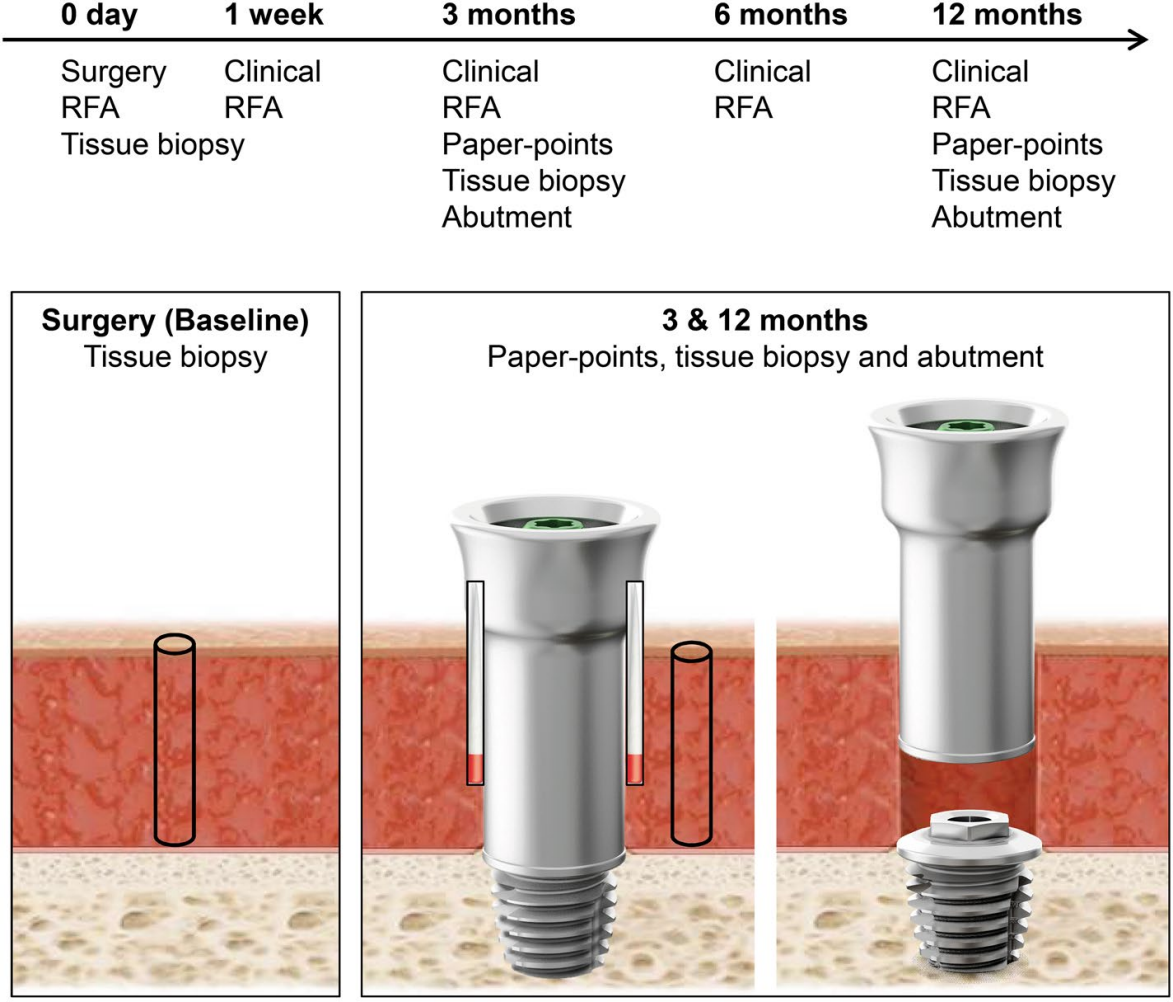
Study outline and schematics of sampling of tissue with biopsy, peri-abutment fluid with paper-points and retrieval of abutment, at baseline, 3 and 12 months. Parallel to the microbiological sampling, clinical measurements were collected and the stability of the implant assessed with resonance frequency analysis (RFA)

Adult patients, eligible for BAHS, were enrolled and the surgery was performed in a tertiary university hospital in Sweden between November 2014 and December 2015. Informed consent was obtained from all participants. Exclusion criteria were (1) inability or unwillingness to participate in follow-up; (2) skin thickness of > 10 mm since soft tissue reduction would then be required; (3) diseases known to compromise bone quality or (4) previous irradiation in the implant area. Enrolled patients were allocated consecutively to the control (machined abutment) and the test (polished abutment) groups. Patients received the Ponto Wide implant (diameter 4.5 mm, length 4 mm), pre-mounted with either a machined or an electro-polished abutment of suitable length (diameter 5 mm, length 6, 9 or 12 mm) (Oticon Medical, Askim Sweden). The implants were installed using the minimally invasive Ponto surgery (MIPS) technique. Here, the drilling and installation is performed through a biopsy incision, eliminating the need for a linear incision and exposure of the bone bed [22, 23]. In brief, the main steps of the MIPS technique are: (1) the site for implant is estimated at 50–55 mm from the ear canal and skin thickness is determined prior to application of local or general anaesthetics; (2) a 5-mm punch biopsy is used for making a circular incision; (3) the cannula is inserted in the circular incision; (4) step-wise drilling is performed through the cannula; (5) the cannula is removed and the implant installed and, finally, (6) a soft healing cap is attached to the abutment and a suitable dressing is applied. After the surgery, the patients were assessed at 5–10 days, 3–12 weeks, 12 weeks, 6 and 12 months following the regular practice of the clinic. In case of adverse soft-tissue reaction, local treatment with Fucidin^®^ ointment containing the antibiotic fusidic acid and corticosteroids (Leo Pharma, Malmo, Sweden) was applied. All data were recorded in paper-based Case Report Forms. A summary of the examinations and parameters is found in Fig. 1.

### Study design

This study was a single-centre, prospective, controlled, case series, clinical investigation. The primary objective was to compare the clinical outcomes and bacterial colonisation between machined and polished abutments 3 months after surgery.

### Abutment design

Abutments (commercially pure titanium grade 4) were either left untreated with a machined surface (control) or electropolished (test) using an ElpoLux TI electrolyte (ElpoChem AG, Volketswil, Switzerland). The inner cavities of the abutment were left untreated by means of masking. Abutments were ultrasonically cleaned stepwise in liquid detergent, deionised water, ethanol and dried in filtered air. All abutments and implants were sterilised by beta-irradiation in a plastic blister.

### Surface characterisation

The chemical composition of the abutment surfaces was analysed using Auger electron spectroscopy (AES) (PHI 700 Scanning Auger Microprobe, 3.0 keV, Physical Electronics Inc., Chanhassen, MN, USA). Scanning electron microscopy (SEM; Leo Ultra 55 FEG-SEM, Zeiss, Oberkochen, Germany) operating at 10 kV was used for qualitative assessment of the surface roughness. Surface topography was obtained using non-contact white light interferometry (WYKO NT9100, Veeco Instruments Inc., Plainview, NY, USA). Surface wettability was determined by contact angle (CA°) measurement at room temperature. A 3-µL drop of deionised water was dispensed on the cylindrical part of the abutment using a syringe and the static CA° was determined using an optical tensiometer (Theta Lite, Biolin Scientific, Gothenburg, Sweden), and calculated with One Attension software (version 2.6, Biolin Scientific).

### Study interventions

At the surgery visit, the implant site was disinfected with ethanol (70% w/v). After local anaesthesia, a skin biopsy was collected at the predetermined retro-auricular position of the abutment using a 1 mm biopsy punch (Fig. 1). The biopsy was preserved in an ESwab™ collection and transport tube containing 1 mL Liquid Amies medium (Copan Italia S.p.A., Brescia, Italy) for baseline bacterial cultures. At the 3 and 12 months follow-up visits, sampling of peri-abutment exudate for bacterial cultures was performed using two paper-points (Roeko ISO, size 45, Coltene, Langenau, Germany) inserted in the space between the abutment and surrounding skin (anterior and posterior locations). They were left for 60 s, pooled and preserved in 1 mL ESwab™ medium for microbiological cultures. Thereafter the abutment was released from the implant. A sterile bottom- and top lid was mounted on the abutment to seal the interior to ensure that the bacterial count reflected only the outer surface of the abutment. The assembly was thereafter placed in 2 mL ESwab™ medium (2 pooled tubes) for subsequent cultures of bacteria. The skin was disinfected and local anaesthesia was administered to the area before installing a new abutment of the same type (test or control). A soft tissue biopsy was collected at the border of the abutment using a 1-mm biopsy punch and preserved in 1 mL ESwab™ medium for subsequent microbiological cultures. Samples were kept submerged at 4 °C before transportation and after arrival to the Microbiology Laboratory (Department of Biomaterials, University of Gothenburg, Sweden) for processing and analysis. Samples were transported at room temperature during 5–24 h.

### Bacteriological evaluation

The soft tissue biopsies, paper-points and retrieved abutments were processed within 2 days after retrieval. Total aerobic and anaerobic bacteria were measured on abutment (CFU/abutment), paper-points (CFU/paper-points) and in soft tissue samples (CFU/biopsy). Additionally, staphylococci, enterococci, *Escherichia coli*, and *Pseudomonas aeruginosa* were quantitated using selective media. The protocol was adapted from UK Standards for Microbiology Investigations [24] and Charalampakis [25] with slight modifications.

#### Homogenisation of samples and dislodging of bacteria

Soft tissue biopsies were homogenised in the ESwab™ medium using a tissue homogeniser (T10 basic, IKA, Staufen, Germany) for 5 min with progressively increased speed. The ESwab™ tube containing two paper-points was vortexed at maximum speed (3200 rpm) for 1 min to dislodge bacteria. To remove the bacteria adherent on the abutments, the ESwab™ tubes containing the abutments were vortexed for 30 s (3200 rpm), followed by 5 min of sonication at 40 kHz and additional vortexing for 30 s.

#### Viability counting (CFU)

A total of sixtenth-dilution series were made in 0.9% saline from each homogenised sample. A volume of 100 µl from dilution 6 (1:1,000,000), dilution 4 (1:10,000), dilution 2 (1:100) and undiluted sample (1:1) was spread on duplicate agar plates of the following media (Media Department, Clinical Microbiology laboratory, Sahlgrenska University Hospital, Sweden): 5% horse blood Columbia agar (H207) for aerobic bacteria, brucella agar (B350) for anaerobic bacteria, staphylococci agar (S316), enterococci agar (E370), and CHROMagar™ Orientation (K181) (CHROMagar, Paris, France). All plates, except brucella, were incubated aerobically at 37 °C and 5% CO_2_ for 2 days until colonies were counted. Brucella plates were incubated under anaerobic conditions (AnaeroJar™ and AnaeroGen™ anaerobic atmosphere generation system; Oxoid Ltd, Hampshire, UK) at 37 °C for 5 days before CFU counting.

A total of 100 µL were transferred from the undiluted homogenised specimen into one TAS (thioglycolate) broth tube (Media Department, Sahlgrenska University Hospital) and incubated under aerobic conditions for 5 days for enrichment and to increase detection. The TAS tube was read (positive/negative growth), and re-plated in case of negative agar cultures.

At least one colony from each positive plate was sub-cultured and stored at − 80 °C.

#### Staphylococcal species identification

One colony was re-suspended in 5 mL 0.9% saline and 100 µL was dispensed in each well of an API® Staph strip (bioMérieux SA, Marcy l’Etoile, France), containing 20 miniature biochemical tests and lysostaphin resistance test, and incubated overnight at 37 °C. After addition of the reagents, the strips were read for their specific colour pattern, generating an identification numerical profile. The APIweb™ software (bioMérieux SA) was used to obtain the organism identification that corresponded to the biochemical or numerical profile of the strip.

### Statistics

Independent t-test was used for the comparison between test and control groups with respect to chemical and topographical surface parameters, mean CFU/abutment, CFU/tissue biopsy, and CFU/paper-points at 3 months, for the comparisons of mean CFU/abutment and CFU/paper-points between time-points (3 and 12 months) as well as for the distribution of Holgers score over observations.

To compare the clinical outcomes between the groups, Mann–Whitney *U* test was performed for continuous variables and Fisher’s exact test for ordered categorical and dichotomous variables. One-way ANOVA followed by Tukey’s post hoc test was employed for the comparison of CFU/tissue biopsy between the three time-points (baseline, 3, and 12 months), and for the comparison between the three sampling methods at each time-point. Pearson correlation was used to evaluate the relationship between clinical and microbiological data, both using test and control groups as separate and pooled. All variables entered in the correlation analysis are described in Online Resource 1.

All statistical analyses were executed using SPSS software (Versions 23.0.0.0, IBM Corporation, USA), and a level of significance of *p* < 0.05 was adopted for all tests.

## Results

### Surface characterisation

AES showed a predominance of Ti, O and C on both abutment surfaces, with minor contamination of Ca, Cl and P. The interferometry analysis of surface roughness on the microscale demonstrated a smoother surface for the polished abutment compared to the machined. Although, both abutment types were hydrophilic, the water contact angle was significantly lower for the polished abutment compared to the machined (mean difference of 18.5 CA°). The complete surface characterisation data are summarised in Online Resource 2.

### Clinical outcome

Baseline characteristics and surgery data were similar between the two study groups (Table 1). Twelve of the included patients who received either a machined (control, *n* = 7) or an electropolished (test, *n* = 5) abutment, and completed the 3 months sampling were included in this analysis.

**Table 1:** Baseline demographics, surgery characteristics and clinical outcome

Baseline demographics	Machined (*n* = 7)	Polished (*n* = 5)	
Age (years); *n* (SD)	64.4 (25.1)	46.2 (24.1)	
Gender; *n* (%)			
Male	4 (57.1)	2 (40.0)	
Female	2 (28.6)	3 (60.0)	
Type of hearing loss; *n* (%)			
Acquired conductive/mixed hearing loss	3 (42.9)	3 (60.0)	
Single-sided deafness	2 (28.6)	1 (20.0)	
Congenital conductive hearing loss	2 (28.6)	1 (20.0)	
Smoking; *n* (%)			
No smoking	7 (100.0)	5 (100.0)	
Smoking	0 (0.0)	0 (0.0)	
Body mass index; *n* (SD)	27.4 (6.2)	29.1 (11.2)	

Surgery characteristics	Machined (*n* = 7)	Polished (*n* = 5)	
Skin thickness; millimetres (SD)	6.4 (1.9)	6.7 (2.6)	
Abutment length; *n* (SD)			
6	1 (14.3)	0 (0.0)	
9	5 (71.4)	3 (60.0)	
12	1 (14.3)	2 (40.0)	

Clinical outcomes per group	Machined	Polished	*p*
Maximum Holgers per patient across all visits 0–12 months; *n* (%)			0.470
Grade 0	5 (71.4)	2 (40.0)	
Grade 1	2 (28.6)	1 (20.0)	
Grade 2	0 (0.0)	1 (20.0)	
Grade 3	0 (0.0)	1 (20.0)	
Grade 4	0 (0.0)	0 (0.0)	
Holgers across all patients and all visits, 0–12 months; *n* (%)			0.168
Grade 0	21 (91.3)	13 (68.4)	
Grade 1	2 (8.7)	3 (15.8)	
Grade 2	0 (0.0)	1 (5.3)	
Grade 3	0 (0.0)	2 (10.5)	
Grade 4	0 (0.0)	0 (0.0)	
ISQ low at baseline; ISQ (SD)	49.6 (10.9)	51.2 (3.3)	0.81
ISQ low increase at 12 months; ISQ (SD)	8.4 (5.7)	2.3 (11.5)	0.54

Clinical outcomes pooled	3 months	12 months	*p*
Holgers; *n* (%)			0.869
Grade 0	9 (75.0)	7 (77.8)	
Grade 1	2 (16.7)	1 (11.1)	
Grade 2	0 (0.0)	1 (11.1)	
Grade 3	1 (8.3)	0 (0.0)	
Grade 4	0 (0.0)	0 (0.0)	
Hygiene; *n* (%)			1.00
None	6 (50.0)	5 (55.6)	
Minimal	4 (33.3)	3 (33.3)	
Moderate	2 (16.7)	1 (11.1)	
Abundant	0 (0.0)	0 (0.0)	
Pain; *n* (%)			0.060
None (VAS = 0)	7 (58.3)	9 (100.0)	
Limited (> 0 VAS ≤ 3)	4 (33.3)	0 (0.0)	
Moderate (> 3 VAS ≤ 7)	1 (8.3)	0 (0.0)	
Extensive (> 7 VAS ≤ 10)	0 (0.0)	0 (0.0)	

*p* values calculated with Fisher’s exact test. Hygiene is defined as amount of debris around the abutment

*ISQ* implant stability quotient

Two implants in the control group were lost during the follow-up period between 3 and 12 months. Clinically relevant adverse skin reactions (Holgers score ≥ 2) were observed for two patients in the test group versus none in the control (Table 1). For one patient, the adverse soft-tissue reactions resulted in removal of the polished abutment. The clinical outcome measures (Holgers score, hygiene, pain, numbness and implant stability; ISQ) did not differ significantly between test and control after 3 and 12 months. The comparisons between 3 and 12 months for each clinical outcome measure were performed on pooled data and did not demonstrate any significant difference (Table 1). Local antibiotic treatment was administered to patients experiencing an adverse soft tissue reaction (three out of seven (43%) in the machined group versus two out of five (40%) in the polished group).

### Bacteriological outcome

**Fig. 2:**
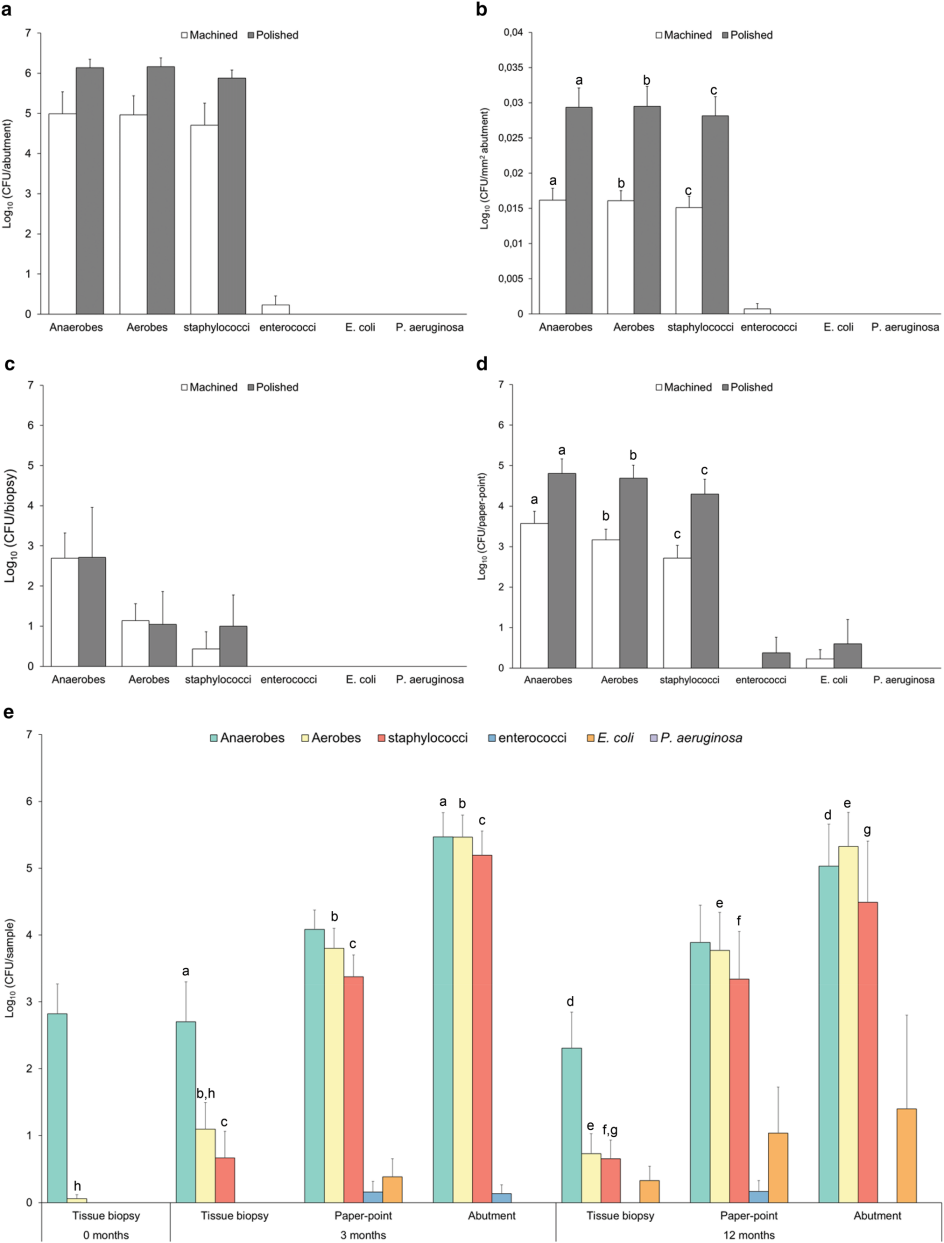
**a**–**e** Viability counts as measured by colony-forming units (CFU) of bacteria adherent on machined and polished abutments (**a, b**), in soft tissues (**c**), and on paper-points (**d**) retrieved at 3 months. **a** CFU per abutment (*n* = 7 machined; and *n* = 5 polished). **b** CFU from abutments normalised by the surface area of the microstructure (*S*_dr_) of the abutment; CFU/mm^2^ (*n* = 7 machined; and *n* = 5 polished). **c** CFU per tissue biopsy (*n* = 12). **d** CFU per paper-point (*n* = 12). **e** Viable counts (CFU/sample type) of aerobic and anaerobic bacteria as well as the aerobic bacterial species (staphylococci, enterococci, *Escherichia coli*, and *Pseudomonas aeruginosa*) at baseline (*n* = 12), 3 months (*n* = 12), and 12 months (*n* = 9 biopsies, *n* = 9 paper-points and *n* = 4 abutments). Data represent mean ± SEM. Bars that share the same letters are significantly different *p* < 0.05

The total viable counts of the evaluated groups of bacteria per abutment (Fig. 2a) or in tissue biopsies (Fig. 2c), were not significantly different between the test and control groups at 3 months. Considering the effect of the additional surface area contributed by the microstructure (*S*_dr_ in Online Resource 2) the total CFU/mm^2^ of anaerobes, aerobes and staphylococci, was significantly higher on the polished abutment surface compared to the rougher machined surface (Fig. 2b).

In the peri-abutment fluid space (paper-points), significantly higher count of anaerobes, aerobes and staphylococci were demonstrated for the test group compared with the control group (Fig. 2d). *Escherichia coli* was found in the fluid space at 3 months (Fig. 2d) and in all three compartments at 12 months (Fig. 2e). At 3 months, enterococci were only retrieved from one machined abutment (Fig. 2a, b) and from paper-points taken from one polished abutment (Fig. 2d). At 12 months enterococci were only isolated from paper-points (Fig. 2e). *Pseudomonas aeruginosa* was not found in any of the compartments at any time-point.

At baseline, the soft tissue was mainly colonised with anaerobic bacteria (Fig. 2e). Anaerobic bacteria were detected in all compartments after 3 and 12 months. The total number of isolated anaerobes was significantly greater on abutments than in tissues at 3 and 12 months.

At baseline, aerobic bacteria were detected in the soft tissue only in one patient (who had the abutment subsequently removed due to adverse skin reactions). At both 3 and 12 months, the total aerobic and staphylococcal viable counts were significantly higher on the abutments compared to the tissue biopsies and paper-points. The number of aerobic bacteria in the tissue biopsies increased significantly between baseline and 3 months (Fig. 2e), whereas no significant temporal change was shown for the rest of samples and bacterial groups.

*Staphylococcus* spp. were not identified at baseline, however found in all compartments after implantation at both 3 and 12 months (Fig. 2e). Biochemical identification of the isolated staphylococcal colonies revealed that several of the patients were colonised by the same species, and probably by the same strain, over the first year of BAHS implantation (Table 2). For five patients, the same species with identical biochemical numerical profile were identified at both time-points. For most patients (10/12), the same species, with identical biochemical numerical profile, was found in at least two of the three compartments at the same time-point. The common skin coloniser *Staphylococcus epidermidis* was identified in all patients but one (11/12), whereas the potentially harmful pathogen *S. aureus* was isolated in five of the patients. S*taphylococcus lugdunensis, Staphylococcus schleiferi* and *Kocuria varians*/*rosea* were isolated in a few patients.

**Table 2:** Staphylococcal species identification of biopsy, paper-point, and abutment samples at 3 and 12 months

Abutment (patient)	3 months			12 months		
	Biopsy	Paper-point	Abutment	Biopsy	Paper-point	Abutment
Machined (101)	*S. aureus* 6736157	*S. aureus* 6736157	*S. aureus* 6736157	*S. epidermidis* 6706116	*S. aureus* 6736157	*S. aureus* 6736157
Machined (102)	NDC	*S. epidermidis* 6706156	*S. epidermidis* 6706156	–	–	–
Machined (103)	*S. epidermidis* 6704116	*S. epidermidis* 6206117	*S. epidermidis* 6206117	*S. schleiferi* 6106145	*S. schleiferi* 6106145	*S. schleiferi* 6106145
Machined (106)	NDC	*S. aureus* 6736157	*S. epidermidis* 6306156	NDC	NDC	*S. aureus* 6736155
Machined (107)	*S. lugdunensis* 6316154	*S. epidermidis* 6706116	*S. epidermidis* 6706116	NDC	*S. lugdunensis* 6316154	*S. epidermidis* 6706156
Machined (108)	*S. epidermidis* 6702157	*S. epidermidis* 6702157	*S. epidermidis* 6702157	–	–	–
Machined (110)	NDC	*S. epidermidis* 6706117	*S. epidermidis* 6706117	*S. epidermidis* 6706117	*S. epidermidis* 6706117	–
Polished (111)	NDC	*S. epidermidis* 6306057	*S. epidermidis* 6306157	NDC	*S. epidermidis* 6306117	–
		*S. lugdunensis* 6716154			*Kocuria varians/rosea* 6104004	
Polished (113)	NDC	*S. epidermidis* 6306117	*S. epidermidis* 6306117	*S. epidermidis* 6306117	*S. epidermidis* 6716157/6716137	–
Polished (114)	NDC	*S. epidermidis* 6706117	*S. epidermidis* 6706117	NDC	*S. epidermidis* 6706157	–
					*S. aureus* 6736157	
Polished (115)	*S. aureus* 6736157	*S. aureus* 6736157	*S. aureus* 6736157	–	–	–
		*S. epidermidis* 6706117	*S. epidermidis* 6706117			
Polished (116)	*S. aureus* 6736157	*S. aureus* 6736157	*S. aureus* 6736157	*S. aureus* 6334056	*S. aureus* 6736157	–

Biochemical numerical profiles using API^®^ Staph strip

– sample not taken, *NDC* no detected colonies on selective staphylococci agar

### Correlation analyses

The correlation analyses revealed several associations between clinical parameters and the microbiological results (Table 3, Online Resource 1). The analysis of specific bacterial groups showed strong positive correlations between Holgers score and pain at 3 months, and Holgers score with aerobic bacteria in the soft tissue at baseline. Similar association was shown for Holgers score at 3 months and tissue anaerobes (3, 12 months). In addition, the CFU counts of all bacterial groups (anaerobes, aerobes, staphylococci, enterococci, *E. coli*) in the peri-abutment fluid space, was positively correlated with Holgers score at 12 months. The hygiene score (amount of debris around the abutment) at 12 months correlated positively with CFU of enterococci isolated from paper-points at 3 months.

**Table 3:** Correlation analysis between clinical and microbiological parameters

Clinical parameters	Microbiological parameters (CFU)	Pooled	
		*r*	*p*
Holgers at 3 m	Tissue aerobes at baseline	0.904	< 0.001
	Tissue anaerobes at 3 m	0.626	0.029
	Tissue anaerobes at 12 m	0.680	0.044
Pain at 3 m	Tissue aerobes at baseline	0.701	0.011
Holgers at 12 m	Paper-point aerobes at 3 m	0.745	0.021
	Paper-point anaerobes at 3 m	0.819	0.007
	Paper-point staphylococci at 3 m	0.705	0.034
	Paper-point enterococci at 3 m	0.884	0.002
	Paper-point *E. coli* at 3 m	0.736	0.024
Hygiene at 12 m	Paper-point enterococci at 3 m	0.746	0.021

Clinical parameters	Clinical parameters	Pooled	
		*r*	*p*
Holgers at 3 m	Pain at 3 m	0.733	0.007
Holgers at 3 m	Hygiene at 12 m	0.737	0.023
Hygiene at 3 m	Hygiene at 12 m	0.763	0.017

Microbiological parameters (CFU)	Microbiological parameters (CFU)	Pooled	
		*r*	*p*
Tissue aerobes at 3 m	Tissue aerobes at 12 m	0.810	0.008
	Tissue staphylococci at 3 m	0.906	0.000
	Tissue staphylococci at 12 m	0.866	0.003
	Paper-point enterococci at 3 m	0.715	0.009
	Paper-point *E. coli* at 3 m	0.895	< 0.001
Paper-point aerobes at 3 m	Abutment aerobes at 3 m	0.729	0.007
	Tissue aerobes at 12 m	0.683	0.043
	Paper-point anaerobes at 3 m	0.868	< 0.001
	Abutment anaerobes at 3 m	0.593	0.042
	Paper-point staphylococci at 3 m	0.898	< 0.001
	Abutment staphylococci at 3 m	0.706	0.010
Abutment aerobes at 3 m	Paper-point anaerobes at 3 m	0.770	0.003
	Abutment anaerobes at 3 m	0.941	< 0.001
	Paper-point staphylococci at 3 m	0.780	0.003
	Abutment staphylococci at 3 m	0.973	< 0.001
Tissue aerobes at 12 m	Tissue anaerobes at 3 m	0.810	0.008
	Tissue staphylococci at 3 m	0.717	0.030
	Tissue staphylococci at 12 m	0.759	0.018
	Paper-point *E. coli* at 3 m	0.698	0.037
Paper-point aerobes at 12 m	Paper-point anaerobes at 12 m	0.974	< 0.001
	Paper-point staphylococci at 12 m	0.976	< 0.001
	Abutment staphylococci at 12 m	0.950	0.050
Tissue anaerobes at 0 m	Abutment enterococci at 3 m	− 0.577	0.050
Paper-point anaerobes at 3 m	Abutment anaerobes at 3 m	0.757	0.004
	Paper-point staphylococci at 3 m	0.894	< 0.001
	Abutment staphylococci at 3 m	0.737	0.006
Abutment anaerobes at 3 m	Paper-point staphylococci at 3 m	0.751	0.005
	Abutment staphylococci at 3 m	0.924	< 0.001
Paper-point anaerobes at 12 m	Paper-point staphylococci at 12 m	0.924	< 0.001
	Paper-point enterococci at 12 m	− 0.718	0.029
Tissue staphylococci at 3 m	Tissue staphylococci at 12 m	0.844	0.004
	Paper-point enterococci at 3 m	0.764	0.004
	Paper-point *E. coli* at 3 m	0.965	< 0.001
Paper-point staphylococci at 3 m	Abutment staphylococci at 3 m	0.768	0.004
	Abutment staphylococci at 12 m	0.952	0.048
Tissue staphylococci at 12 m	Paper-point *E. coli* at 3 m	0.798	0.010
Paper-point staphylococci at 12 m	Abutment staphylococci at 12 m	0.998	0.002
Paper-point enterococci at 3 m	Paper-point *E. coli* at 3 m	0.873	< 0.001
Tissue *E. coli* at 12 m	Paper-point *E. coli* at 12 m	0.996	< 0.001

The data show clinical parameters that revealed significant positive or negative correlations with bacterial count in the three different compartments (in the tissue, in the peri-implant exudate and on the abutment). Pearson coefficients (*r*) and level of significance (*p*) are presented; *n* = 9–12. In the analysis patients with both types of abutments were pooled

For the clinical parameters, a positive correlation was demonstrated between Holgers score at 3 months and the reported pain score at the same time-point (Table 3). Further, the hygiene at 12 months was correlated with the Holgers score, 3 months after surgery as well as with the hygiene after 3 months.

For the microbiological parameters, at 3 months paper-point sampling of aerobes, anaerobes and staphylococci correlated with abutment sampling of aerobes, anaerobes and staphylococci, respectively (Table 3). At 12 months, there was an association between paper-point and abutment CFU of staphylococci, and between tissue and paper-point sampling of *E. coli*.

## Discussion

### Clinical outcome

The present study could describe the bacterial flora on the abutment, in the surrounding exudate and in the peri-abutment soft tissue from baseline up to 12 months. Clinical parameters such as Holgers score, ISQ, hygiene, pain or numbness were in accordance with previous data for BAHS installed using tissue preservation techniques [23, 26] and no difference between polished and machined abutments were found.

Discrepancy of the elastic modulus between the abutment and the soft tissue produces interfacial strain concentrations, leading to micro-trauma and cell activation, resulting in a constant inflammatory state of the peri-abutment skin [15, 27]. The peri-abutment skin moves both passively when moving the head and actively because of facial movements or external forces [23]. By integrating the rigid metal abutment with the mobile soft tissue, as with the hydroxyapatite-coated abutments, the strains might increase further. Here, we hypothesised that by preventing soft tissue adhesion and integration, the soft tissue can move more freely around the abutment, counteracting the tissue strains. Moreover, a smooth abutment surface might reduce debris build-up, which may attract microbial colonisation, as well as being easier to clean. Improved clinical outcome could, however, not be revealed for the polished abutment using the conventional macroscopical clinical outcome measures. Other techniques, such as gene expression and morphology may be alternative tools to determine the impact of skin movements and strain.

### Bacteriological outcome

In this study, presence of viable bacteria in all three compartments was shown. Little is known about the distribution of bacteria on and around abutments of BAHS in patients with or without peri-abutment skin infection. Although polymicrobial biofilms on fixtures, abutments, and internal screws have been detected [7], ultrastructural observations failed to detect bacteria directly adherent on the abutment surface [6]. Still, an implant surface may harbour microbes and elicit infection in the surrounding soft tissues. This is supported by experimental observations of a higher expression of pro-inflammatory genes at infected versus non-infected sites as well as a higher TLR2 expression (indicative of bacteria–host cell interactions) close to a colonised implant surface than further out in tissue [28].

The total number of viable bacteria on the abutment was similar for the two abutment types, however more were present on the polished if normalised to the microroughness. Even though surface topography is known to influence how microorganisms adhere and form a biofilm [29], in this study the microtopography of the two abutments was possibly too similar. However, higher CFU counts were revealed in the peri-abutment fluid for the polished group compared with the machined group. A plausible explanation is that the structural barrier between the soft tissue and the polished surface is more permissive for the downward migration of bacteria. To achieve a successful soft-tissue integration, fibroblasts must win the ‘race for the surface’ against microorganisms on the implant surface [10, 30]. Against the assumption that a tight structural barrier is a prerequisite, are the recent findings that tissue-integrating hydroxyapatite-coated abutments were still at risk for infection [14, 31].

How the biological processes elicited around skin-penetrating implants are related to the presence of a specific biomaterial surface morphology and microorganism is largely unknown [6, 32], and in vitro studies demonstrate contrasting results when comparing rough and smooth surfaces [33, 34]. Infection remains a challenge for porous polyethylene used for reconstruction or augmentation in the cranio-maxillo-facial region [35]. Possible factors for these infections could be a reduced tissue infiltration and vascularisation in the porous structure, allowing microorganisms to evade host defence mechanisms [36].

In this study, the surface topography did not affect the CFU count in the soft tissue at 3 months. The biopsies were taken a few millimetres away from the abutment, and the closer to an implant the more the material influences the biological and microbiological processes [32, 37]. Independent of infection or not, studies have shown a concentration of inflammatory cells superficial to the skin compared to deeper; and less inflammatory cells further away from the abutment [38, 39].

The skin is not sterile and is colonised by numerous bacteria, which are also found in deeper layers of the skin [40]. Skin disinfection before surgery removes transient superficial bacteria and reduces resident bacteria in the deeper layers. Therefore, whereas transient bacteria are easily removed, commensals are difficult to eradicate completely and can cause infections if allowed to multiply [41]. Surgical site infections can be diagnosed by the presence of clinical symptoms alone, or with more than 10^6^ CFU of skin commensals per mm^3^ tissue, overcoming host’s defences [42]. In this study, while abutments were colonised by 10^3^–10^6^ CFU, the mean CFU from skin biopsies was below that level. Interestingly, for the patient with Holgers score 3 and pain score 3.5 at 3 months, there was a presence of tissue aerobes at surgery (5 CFU/biopsy) and tissue anaerobes at 3 months (1.9 × 10^6^ CFU/biopsy), the highest total viable counts at these time-points.

Colonisation during implant surgery, post-operative soft tissue infection, and haematogenous seeding are the main studied routes of implant-associated infection. In contrast, the infection route from within the soft tissue towards the percutaneous abutment has barely been studied [43]. An important finding in the present study was that anaerobic bacteria were already present in the tissue prior to BAHS installation despite disinfection of the skin surface. Furthermore, over time both anaerobic and aerobic bacteria were present in the soft tissue. Hence, microbes within the soft tissue could provide a route for bacterial colonisation of the abutment, a possibility demonstrated for catheter-associated infection by others [32]. The particular and multi-species microbiota, possibly found as biofilm communities on and around the abutment, provides a plausible explanation for the clinical recurrent or persistent inflammation and infection [7]. Nevertheless, colonisation per se does not necessarily imply infection, although it represents the first step of microbial infection by the accession and establishment of pathogens to the site [44]. Therefore, while colonisation is the presence of bacteria on a body surface without causing disease, infection is the invasion of host tissues by microorganisms resulting in disease [31]. The remaining question is which is the minimal infectious dose and the pathogen. Clinical guidelines for the treatment of microbial colonisation states are limited, due to a lack of knowledge of the pathogenesis of colonisation and the host–microbe relationship aspects that influence the development of infection [45]. Based on the observations of anaerobic bacteria in all three compartments evaluated, it is evident that future studies should include the detection of anaerobes to elucidate their role(s) in the microbe–host symbiosis and/or BAHS-associated infection. Staphylococci have been the most common pathogen in association with failed, retrieved BAHS fixtures and their internal screws [7]. In this study, *Staphylococcus* spp. were also the most commonly isolated aerobic genus across all samples and time-points. It is well known that *S. aureus* constitutes a risk factor for the development of skin, soft tissue, and systemic infections [19, 46], and in this study it was detected in 5/12 patients. This is in agreement with the detection of *S. aureus* in 47% of patients with percutaneous bone-anchored lower limb amputation prostheses [47].

### Associations between clinical and microbiological outcomes

Although the use of the Holgers score has been questioned recently [47, 48], the present data indicate that there is an association between Holgers score and the number of bacteria in the peri-abutment fluid and surrounding tissue, as well as with pain scores. While *P. aeruginosa* was not detected, 4 patients were colonised by *E. coli* of which one was colonised at both 3 and 12 months. The isolation of *E. coli* from the peri-implant fluid space at 3 months correlated with an increased Holgers score at 12 months. In general, Gram-negative bacteria are not part of the skin microbiota, and their detection in skin samples is probably due to contamination from the gastrointestinal tract [49]. These findings stress the need for efficient disinfection before implantation and the implementation of an efficient patient-cleaning regimen. The associations between the amount of debris around the abutment at 3 and 12 months, between Holgers score at 3 months and amount of debris later at 12 months, and between Holgers score at 12 months and CFU counts from most bacterial species retrieved by paper-points at 3 months, indicate the importance of the individual compliance with the cleaning regimen.

### Sampling methods and strength and limitations of study

Although genomic approaches to characterise the skin bacterial flora have shown a much greater diversity of organisms than culture-based methods, the latter are the standard use in clinical microbiology for the detection and quantification of bacteria in samples. Genomic approaches cannot provide information if the bacterial species found are alive or viable like with culturing, although it is also known than certain species are difficult to culture and may go undetected. To increase bacterial detection, additional enrichment of the samples in TAS media for additional 5 days was performed. In this study, selective media to evaluate *Propionibacterium* spp were not included. Fungal-associated dermatologic diseases could also play a role for BAHS [50]; however, fungi and viruses were not evaluated from the obtained samples.

Sampling from the three compartments enabled studies on the relationship between clinical macroscopical observations and microbiological findings. To our knowledge, this is the first time in the field of BAHS where the microbiological status in the tissue, in the space between abutment and tissue, and on the abutment, is evaluated over time.

Prior to acquiring knowledge on the mechanism of BAHS-associated infection, it is not possible to advocate a specific compartment as more important for the microbiological sampling. The paper-point sampling has definite advantages as being non-invasive, and allowing standardisation. Moreover, this method was found to provide the largest number of correlations with other parameters at 3 months (paper-point > tissue > abutment). The soft-tissue biopsy enabled quantitative CFU determinations, but the variation in biopsy size presents an obvious difficulty with normalisation and intra- and inter-patient comparisons (between compartments and between patients). Sampling bacteria from the abutment suffers the weakness of a necessary removal of the abutment. For example, due to a more complex and more invasive approach than what was anticipated, the abutment removal and sampling at 12 months was discontinued (resulting in fewer abutment samples at this time-point).

## Conclusions and clinical implications

- The modification of the abutment topography did not affect the clinical outcome during the first year of a BAHS.
- Sampling from three different compartments (soft tissue biopsy, peri-abutment fluid space and abutment) allowed the isolation and quantitative determination of the number of viable bacteria.
- Several correlations were found between the clinical and the microbiological findings.
- The detection of anaerobic bacteria in skin biopsies prior to BAHS installation and subsequently in all three compartments, motivates further studies on their characterisation and putative role in the BAHS-associated microbiome and infection.
- In the peri-abutment space exclusively, a significantly higher amount of anaerobes, aerobes and staphylococci were demonstrated for polished versus machined abutments.
- Biochemical identification of staphylococci showed that the skin commensal *S. epidermidis* and pathogen *S. aureus* were common species. The presence of *S. aureus* represents a risk factor for the development of BAHS-associated infections.

Taken together, ahead of a large clinical trial, the present pilot trial largely confirmed a suitable study design, sampling and analytical methodology to determine the effects of modified abutment properties.

## Electronic supplementary material

Below is the link to the electronic supplementary material.

Supplementary material 1 (PDF 238 KB)

Supplementary material 2 (DOCX 3080 KB)

Supplementary material 3 (DOCX 19 KB)
