# Supplementary material for: The clinical outcome and microbiological profile of bone-anchored hearing systems (BAHS) with different abutment topographies: a prospective pilot study
Source: Eur Arch Otorhinolaryngol. 2018 Apr 5;275(6):1395–408. doi: 10.1007/s00405-018-4946-z (PMC5951894; doi:10.1007/s00405-018-4946-z)
Supplement: Supplementary file 1 — Supplementary material 1 (PDF 238 KB) [file 405_2018_4946_MOESM1_ESM.pdf]

Online Resource 1. Biospecimen Reporting for Improved Study Quality (BRISQ): Items to Consider Reporting if Known and Applicable (Moore HM, Kelly AB, Jewell SD, et al. Biospecimen reporting for improved study quality (BRISQ). Cancer cytopathology. Apr 25 2011;119(2):92-101)

Apply to	Tier #	Item Description	Item #	Location
**I. Pre-acquisition**				
All	Tier 1	**Biospecimen type. Solid tissue, whole blood, serum/plasma, isolated cells, urine, secretions, or another product derived from a human being.**	Tissue, Abutment, Exudate	p. 5, 8, 9________
All	Tier 1	**Anatomical or collection site. In standard terminology, organ(s) of origin or site of blood draw.**	Retroauricular area	p. 7______________
All	Tier 1	**Biospecimen disease status. From controls or individuals with the disease of interest; in the case of solid tissue, whether it is from disease site or normal adjacent (not involved but from the same anatomical site as a disease specimen in the same patient).**	Biospecimens from (i) titanium surface, (ii) exudate surrounding titanium surface, (iii) soft tissue biopsy 5 mm from titanium, at the treatment site	p. 7______________
All	Tier 1	**Clinical characteristics of patients. In standard terminology, available medical information known or believed to be pertinent to the condition of the biospecimens.**	Hearing deficiency	p. 5, Table 1______
All	Tier 1	**Vital state. Alive or deceased when biospecimens were obtained**	Alive	p. 5______________
All	Tier 3	*Disease state. Patient condition relative to disease and treatment, if known (eg, during- or post-therapy; acute, chronic, or terminal stage).*	Chronic	p. 5, Table 1_______
All	Tier 3	*Cause of death. For postmortem biospecimens, the cause of death and other diseases present at the time of death.*	Not applicable	_________________
All	Tier 3	*Agonal state. The patients' physical condition immediately preceding death (eg, prolonged degeneration or relatively healthy)*	Not applicable	_________________
All	Tier 1	**Diagnosis. Patient diagnoses pertinent to the study being conducted, using an accepted system of standards (eg, the Systemized Nomenclature of Medicine or the International Classification of Diseases). Please note that clinical and pathology diagnoses are not always the same.**	Congenital conductive hearing loss, acquired conductive-mixed hearing loss, single sided deafness	Table 1___
All	Tier 1	**Clinical. Patient clinical diagnoses (determined by medical history, physical examination, and analyses of a biospecimen) pertinent to the study being conducted.**	Congenital conductive hearing loss, acquired conductive-mixed hearing loss, single sided deafness	Table 1___
All	Tier 1	**Pathology. Patient pathology diagnoses (determined by macro and/or microscopic evaluation of a biospecimen at the time of diagnosis and/or prior to research use) pertinent to the study being conducted.**	Not applicable	_________________
All	Tier 2	Time between diagnosis and sampling. The time or range of time between disease diagnosis and sample acquisition.	Time between treatment and sampling: 0, 3, 12 months	p. 7 _________
All	Tier 3	*Exposures. Neoadjuvant therapy, other current or past medical treatments or environmental factors that might influence the condition of the biospecimen (eg, chemo-and radiation therapy, blood thinner, smoking status).*	Exclusion criteria, demographics	p. 7, Table 1
All	Tier 3	*Reproductive status. The hormonal or reproductive state of the patients (eg, pregnant, pre-pubescent, post-menopausal).*	Not applicable	_________________
All	Tier 2	Patient demographic information. Demographic information that might be relevant to the condition of the biospecimens (eg, age range, gender).	Demographics	Table 1___________
All	Tier 2	Accrual scheme. Whether the biospecimens were obtained for the study being conducted or for a generalized collection such as a population-based biospecimen resource (i.e. retrospective or prospective procurement); whether any standard operating procedures (SOPs) were employed and whether these SOPs are available to others upon request. Reference any clinical trials relevant to the accrual scheme.	Obtained for the study; sampling SOPs available	p. 7__________
All	Tier 2	Nature of the biobanking institution(s). The biobanking context in which the biospecimens were obtained (eg, as part of an internal collection or a biospecimen-acquisition network); include name, location, and primary contact details such as email address or Web site and reference to any pertinent SOPs.	Biobank 513	p. 7_________________
**II. Acquisition**				
All	Tier 1	**Collection mechanism and parameters. How the biospecimens were obtained (eg, fine needle aspiration, pre-operative blood draw).**	Tissue biopsy punch; retrieved abutment; paper-point absorption of exudate	p. 7 _________________
Tissue	Tier 3	*Time from cessation of blood flow in vivo to biospecimen excision/acquisition. The time or range of times that the biospecimens were ischemic in the body.*	Not applicable	_________________

(Continued)

Online Resource 1. (Continued)

Apply to	Tier #	Item Description	Item #	Location
All	Tier 2	Time from biospecimen excision/acquisition to stabilization. The time or time-range between when the biospecimens were obtained (eg, blood drawn or tumor surgically removed) and when they were stabilized. *For postmortem biospecimens*, list the postmortem interval range (i.e. the time from death to stabilization of the biospecimen).	Immediately	p. 7
All	Tier 2	Temperature between biospecimen excision/acquisition and stabilization. The temperature or range thereof at which biospecimens were kept between when biospecimens were obtained (eg, blood drawn or tumor surgically removed) and when they were stabilized. *For postmortem biospecimens*, the temperature at which the cadaver was stored during the postmortem interval.	Room temperature	p. 7
Fluid	Tier 2	Collection container. The kind of tube into which biospecimens were captured as they left the body.	ESwab™	p. 7
**III. Stabilization/Preservation**				
All	Tier 1	**Mechanism of stabilization. The initial process by which biospecimens were stabilized during collection [eg, snap or controlled-rate freezing, fixation, additive (heparin, citrate, or EDTA), none].**	Total immersion in liquid Amies medium	p. 7
All	Tier 1	**Type of long-term preservation. The process by which the biospecimens were sustained after collection (eg, freezing and at which temperature; formalin fixation, paraffin embedding; additive; none). Please note, this might or might not differ from the mechanism of stabilization.**	Total immersion in liquid Amies medium	p. 7
All	Tier 1	**Constitution and concentration of fixative/preservation solution. The make-up of any formulation employed to maintain the biospecimens in a non-reactive state (eg, 10 percent neutral-buffered formalin or 10 USP Heparin Units/mL).**	Liquid Amies medium	p. 7
Tissue	Tier 2	Time in fixative/preservation solution. The time or range thereof that biospecimens were exposed to the preservation medium.	1-2 days	p.7, 8
Tissue	Tier 2	Temperature during time in preservation solution. The temperature of the medium during the preservation process.	at 4°C and room temperature	p. 7
Fluid	Tier 2	Aliquot volume. The amount in each liquid biospecimen sample.	Absorbed in 2 Roeko size 45 paper-points	p. 7
Tissue	Tier 2	Specimen size. The approximate size or weight of solid biospecimen samples processed (eg, cubes approximately 0.5 cm on a side, 0.5 gram).	1 mm punch	p. 7
**IV. Storage/Transport**				
		**Storage parameters. The conditions under which the biospecimens were maintained until analysis.**		
All	Tier 1	**Storage temperature. The temperature or range thereof at which the biospecimens were maintained until distribution or analysis.**	4°C or room temprerature	p. 7, 8
All	Tier 1	**Storage duration. The time or range thereof between biospecimen acquisition and distribution or analysis.**	Maximum 2 days	p. 7, 8
All	Tier 2	Storage details. Other conditions under which specimens were maintained during storage (eg, to minimize oxidation).	Samples completely submerged	p. 7
All	Tier 3	*Type of storage container. The vessel in which biospecimens were kept.*	Plastic Tube	p. 7
All	Tier 3	*Type of slide. The microscope slides to which biospecimens were affixed.*	Not applicable	
		**Shipping parameters. The conditions to which biospecimens were exposed during each shipment or inventory management.**		
All	Tier 1	**Shipping temperature(s). The temperature or range thereof at which biospecimens were maintained during each shipment or relocation.**	Room temperature	p. 7, 8
All	Tier 2	Shipping duration. The time, estimate, or range thereof that the biospecimens spent in shipment each time they were transported.	5-24 h	p. 7, 8
All	Tier 3	*Type of transport container. The type of vessel (eg, pre-manufactured shipping container, polystyrene box) and the packing material in which the biospecimens were transported.*	Carton box	p. 7
All	Tier 3	*Shipping parameters. Other conditions under which the biospecimens were transported (eg, vacuum sealing, desiccant, packing material). Please note any deviations from standard operating procedures that might influence the condition of the biospecimens (eg, shipping anomalies that exposed paraffin blocks to high temperatures).*	Carton box with an upright position of the tubes	p. 7
		Freeze-thaw parameters. The conditions to which biospecimens were subjected during any thaw events.		

(Continued)

Online Resource 1. (Continued)

Apply to	Tier #	Item Description	Item #	Location
Fluid	Tier 2	Number of freeze-thaw cycles. The number, estimate, or range thereof of thaw-refreeze events to which biospecimens were subjected prior to analysis.	Not applicable	________________
Fluid	Tier 3	*Duration of thaw events. The amount of time or range thereof the biospecimens spent thawed prior to the final thaw before processing.*	Not applicable	________________
Fluid	Tier 3	*Time from last thaw to processing. The time or range of times between unfreezing and analysis.*	Not applicable	________________
All	Tier 3	*Temperature between last thaw and processing. The temperature at which biospecimens were kept between unfreezing and analysis.*	Not applicable	________________
**V. Quality Assurance Measures Relevant to the Extracted Product and Processing Prior to Analyte Extraction and Evaluation**				
All	Tier 1	**Composition assessment and selection. Any parameters that were used to evaluate and/or choose biospecimens for inclusion in the study.**	Visual inspection	p. 8______________
All	Tier 2	Gross and microscopic review. The anatomical characteristics of the biospecimens in the study and the relevant qualifications of the individual performing the review (eg, anatomist, pathologist, hematologist, microbiologist, or researcher).	Visual inspection by a microbiologist (MT)	________________
Tissue	Tier 2	Proximity to primary pathology of interest. Whether the biospecimen was taken from a region adjacent to or distal from another region of interest, such as a tumor or area of necrosis. Give approximate distances if known.	All specimens taken from the immediate site of interest. Soft tissue biopsy (3 m, 12 m) obtained 5 mm from abutment	p. 7_____________
All	Tier 2	Method of enrichment for relevant component(s). The method by which pertinent portions of the biospecimen were separated from the rest of the biospecimen (eg, laser-capture microdissection of tissue, block selection for region of lesion, centrifugation of blood).	Cultures in thioglycolate broth	p. 9_____________
All	Tier 2	Details of enrichment for relevant component(s). The parameters used to separate pertinent portions of the biospecimen from the rest of the biospecimen, if applicable (eg, centrifugation speed and temperature).	5 days additional enrichment	p. 9___________
Tissue	Tier 3	*Embedding reagent/medium. Any formulation used to enclose the biospecimens (eg, paraffin).*	Not applicable	________________
All	Tier 2	Quality assurance measures. Any methods used to assess the quality of the biospecimens relevant to the biomolecular analyte, when these methods were employed (eg, prior to long-term storage or immediately before experimental analysis), and the results (eg, RNA integrity number, hemolysis assessment).	Testing of selective media with relevant control strains	p. 9____________

Bold: Tier 1–Recommended to report.
Plain: Tier 2–Beneficial to report.
Italics: Tier 3–Additional items to report.

The clinical outcome and microbiological profile of bone anchored hearing systems (BAHS) with different abutment topographies – A prospective pilot study

Margarita Trobos[1Δ], Martin Lars Johansson[1,2Δ], Sofia Jonhede[2], Hanna Simonsson[2], Maria Hoffman[1], Omar Omar[1], Peter Thomsen[1], Malou Hultcrantz[3]

[1]Department of Biomaterials, Institute of Clinical Sciences, Sahlgrenska Academy, University of Gothenburg, Gothenburg, Sweden

[2] Oticon Medical AB, Askim, Sweden

[3] Department of Otorhinolaryngology, Karolinska University Hospital, Stockholm, Sweden

[Δ] These authors contributed equally to this work.

Corresponding author: Margarita Trobos

Address: Department of Biomaterials, Institute of Clinical Sciences, Sahlgrenska Academy, University of Gothenburg

P.O. Box 412

405 30 Gothenburg, Sweden

Email: margarita.trobos@biomaterials.gu.se
