# Supplementary material for: The clinical outcome and microbiological profile of bone-anchored hearing systems (BAHS) with different abutment topographies: a prospective pilot study
Source: Eur Arch Otorhinolaryngol. 2018 Apr 5;275(6):1395–408. doi: 10.1007/s00405-018-4946-z (PMC5951894; doi:10.1007/s00405-018-4946-z)
Supplement: Supplementary file 2 — Supplementary material 2 (DOCX 3080 KB) [file 405_2018_4946_MOESM2_ESM.docx]

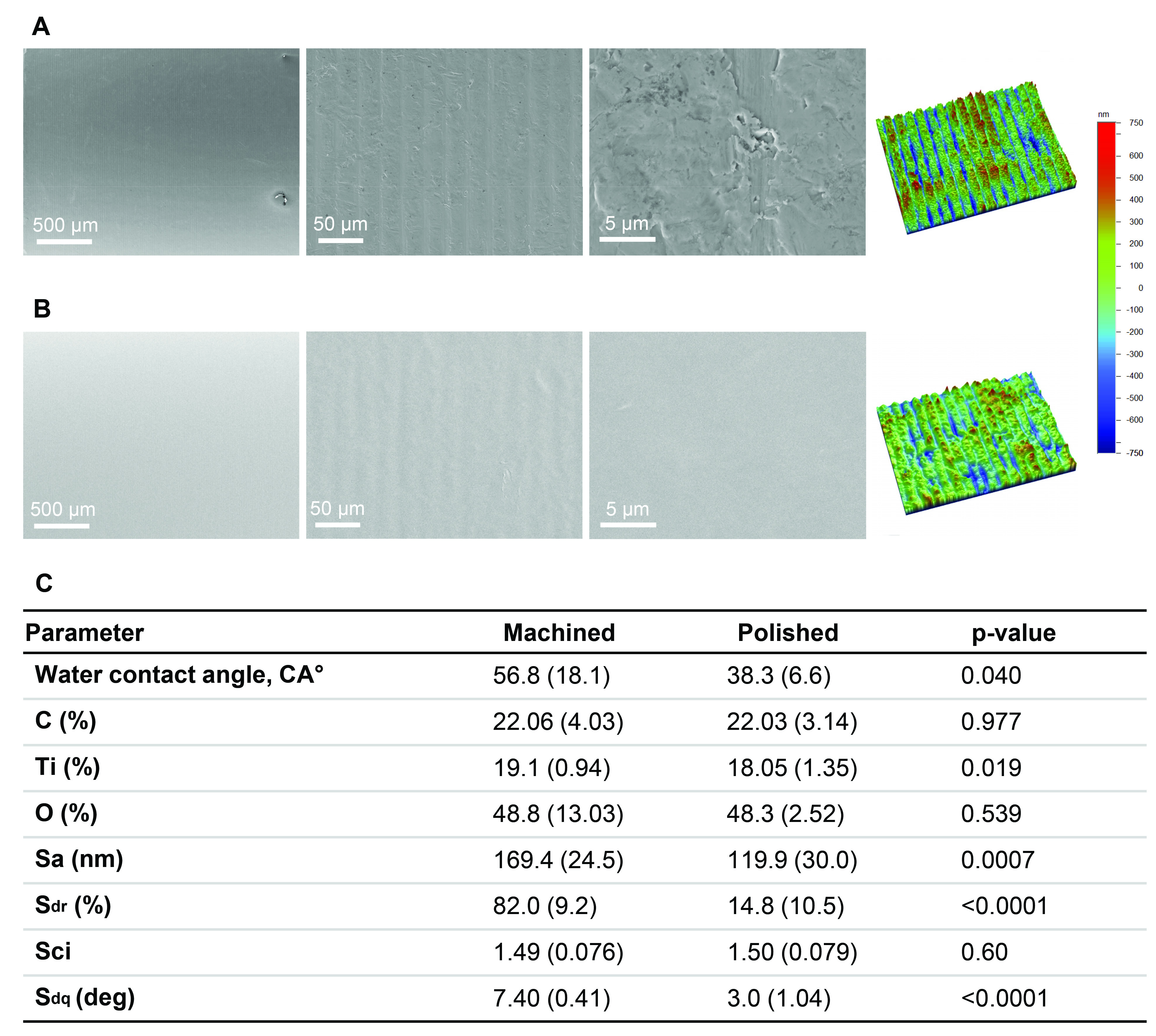


**Online Resource 3.** **Qualitative and quantitative topographical and chemical characterisation of the machined and polished abutments.** Scanning electron micrographs of the machined (A) and the polished (B) abutment at different resolutions (100x, 1 000x and 5 000x). Surface microtopography of the two different abutment types is also shown. The medium resolution images show the typical banded feed marks in the form of ridges and grooves created during turning on both types of abutment, however for the polished abutments these ridges were rounded off (B). In higher magnification, the surface of the machined implants is characterized by visible grooves, tearing and smeared material caused by the turning process. On the polished abutments, these features were not visible, instead it was characterised by a smooth and shiny surface. (C) The water contact angle, surface elemental composition determined by AES and topographical parameters determined by interference microscopy. All data presented as mean values (SD). Surface topography CA°-values presented are mean from three abutments of each type. AES-values presented are mean from four abutment samples of each type, with four measurements on each abutment. Data are normalised to consider only the elements C, Ti and O. Surface topography measurements were performed using a non-contact white light interferometry system, Veeco WYKO NT9100. Mean values from measurements on three machined abutments samples and four polished abutment samples. On each sample, the topography was measured in three 310x235μm areas. Sa: arithmetic mean deviation of the surface; Sdr: developed surface area ratio; Sci: surface core fluid retention index; Sdq: root mean square gradient. Data were processed by extrapolation of “invalid pixels” (modulation threshold was set to 3%) and tilt plus cylinder shape correction. Data were smoothed by a 3x3 median filter to reduce noise

**The clinical outcome and microbiological profile of bone anchored hearing systems (BAHS) with different abutment topographies – A prospective pilot study**

Margarita Trobos^1∆^, Martin Lars Johansson^1,2∆^, Sofia Jonhede^2^, Hanna Simonsson^2^, Maria Hoffman^1^, Omar Omar^1^, Peter Thomsen^1^, Malou Hultcrantz^3^

^1^Department of Biomaterials, Institute of Clinical Sciences, Sahlgrenska Academy, University of Gothenburg, Gothenburg, Sweden

^2^ Oticon Medical AB, Askim, Sweden

^3^ Department of Otorhinolaryngology, Karolinska University Hospital, Stockholm, Sweden

^∆^ These authors contributed equally to this work.

**Corresponding author:** Margarita Trobos

Address: Department of Biomaterials, Institute of Clinical Sciences, Sahlgrenska Academy, University of Gothenburg

P.O. Box 412

405 30 Gothenburg, Sweden

Email: margarita.trobos@biomaterials.gu.se
