# Supplementary material for: The clinical outcome and microbiological profile of bone-anchored hearing systems (BAHS) with different abutment topographies: a prospective pilot study
Source: Eur Arch Otorhinolaryngol. 2018 Apr 5;275(6):1395–408. doi: 10.1007/s00405-018-4946-z (PMC5951894; doi:10.1007/s00405-018-4946-z)
Supplement: Supplementary file 3 — Supplementary material 3 (DOCX 19 KB) [file 405_2018_4946_MOESM3_ESM.docx]

**Online Resource 2.** The different variables and the number of patients (n) in the correlation analyses

|  | **Correlated variables** | **Data variable** | **Unit of measurement**  **(n=number of patients)** |
| --- | --- | --- | --- |
| Bacteria | Tissue Aerobes at baseline | Continuous | CFU (n = 12) |
|  | Tissue Anaerobes at baseline | Continuous | CFU (n = 12) |
|  | Tissue staphylococci at baseline | Continuous | CFU (n = 12) |
|  | Tissue enterococci at baseline | Continuous | CFU (n = 12) |
|  | Tissue *E. coli* at baseline | Continuous | CFU (n = 12) |
|  | Tissue Aerobes at 3 months | Continuous | CFU (n = 12) |
|  | Tissue Anaerobes at 3 months | Continuous | CFU (n = 12) |
|  | Tissue staphylococci at 3 months | Continuous | CFU (n = 12) |
|  | Tissue enterococci at 3 months | Continuous | CFU (n = 12) |
|  | Tissue *E. coli* at 3 months | Continuous | CFU (n = 12) |
|  | Tissue Aerobes at 3 months | Continuous | CFU (n = 12) |
|  | Tissue Anaerobes at 12 months | Continuous | CFU (n = 12) |
|  | Tissue staphylococci at 12 months | Continuous | CFU (n = 12) |
|  | Paper-point Aerobes at 3 months | Continuous | CFU (n = 12) |
|  | Paper-point Anaerobes at 3 months | Continuous | CFU (n = 12) |
|  | Paper-point staphylococci at 3 months | Continuous | CFU (n = 12) |
|  | Paper-point enterococci at 3 months | Continuous | CFU (n = 12) |
|  | Paper-point *E. coli* at 3 months | Continuous | CFU (n = 12) |
|  | Paper-point Aerobes at 12 months | Continuous | CFU (n = 12) |
|  | Paper-point Anaerobes at 12 months | Continuous | CFU (n = 12) |
|  | Paper-point staphylococci at 12 months | Continuous | CFU (n = 12) |
|  | Paper-point enterococci at 12 months | Continuous | CFU (n = 12) |
|  | Paper-point *E. coli* at 12 months | Continuous | CFU (n = 12) |
|  | Abutment Aerobes at 3 months | Continuous | CFU (n = 12) |
|  | Abutment Aerobes per mm^2^ at 3 months | Continuous | CFU (n = 12) |
|  | Abutment Anaerobes 3 months | Continuous | CFU (n = 12) |
|  | Abutment Anaerobes per mm^2^ at 3 months | Continuous | CFU (n = 12) |
| Clinical parameters | Holgers at 3 months | Categorical (Ordinal) | 0-4 (n=12) |
|  | Holgers at 12 months | Categorical (Ordinal) | 0-4 (n=9) |
|  | Pain at 3 months (grouped VAS score) | Categorical (Ordinal) | 0-3 (n=12) |
|  | Pain at 12 months (grouped VAS score) | Categorical (Ordinal) | 0-3 (n=9) |
|  | Hygiene at 3 months | Categorical (Ordinal) | 0-3 (n=12) |
|  | Hygiene at 12 months | Categorical (Ordinal) | 0-3 (n=9) |

**The clinical outcome and microbiological profile of bone anchored hearing systems (BAHS) with different abutment topographies – A prospective pilot study**

Margarita Trobos^1∆^, Martin Lars Johansson^1,2∆^, Sofia Jonhede^2^, Hanna Simonsson^2^, Maria Hoffman^1^, Omar Omar^1^, Peter Thomsen^1^, Malou Hultcrantz^3^

^1^Department of Biomaterials, Institute of Clinical Sciences, Sahlgrenska Academy, University of Gothenburg, Gothenburg, Sweden

^2^ Oticon Medical AB, Askim, Sweden

^3^ Department of Otorhinolaryngology, Karolinska University Hospital, Stockholm, Sweden

^∆^ These authors contributed equally to this work.

**Corresponding author:** Margarita Trobos

Address: Department of Biomaterials, Institute of Clinical Sciences, Sahlgrenska Academy, University of Gothenburg

P.O. Box 412

405 30 Gothenburg, Sweden

Email: margarita.trobos@biomaterials.gu.se
